# Expression of metabolic genes in NK cells is associated with clinical outcomes in patients with severe COVID-19: a brief report

**DOI:** 10.3389/fcimb.2025.1636463

**Published:** 2025-08-25

**Authors:** Kenia Y. Osuna-Espinoza, Manuel G. Mejia-Torres, Adrian Camacho-Ortiz, Eduardo Perez-Alba, Azalia M. Martinez-Castilla, Mario C. Salinas-Carmona, Adrian G. Rosas-Taraco

**Affiliations:** ^1^ Universidad Autónoma de Nuevo León, Servicio y Departamento de Inmunología, Facultad de Medicina, Monterrey, NL, Mexico; ^2^ Universidad Autónoma de Nuevo León, Servicio de Infectología, Facultad de Medicina y Hospital Universitario “Dr. José Eleuterio González”, Monterrey, NL, Mexico

**Keywords:** severe COVID-19, NK cells, metabolism, SIRT1, AMPK, HIF1A, GLUT1

## Abstract

Natural killer (NK) cells are innate lymphocytes with cytotoxic activity against tumors and viruses. The pandemic of the coronavirus disease 2019 (COVID-19) has increased the investigation of their role in disease severity. However, their functional status and modulators remain controversial. Recent studies highlighted the role of metabolism in immune function, but metabolic changes in NK cells during SARS-CoV-2 infection remain unexplored. This study compares metabolic (*SIRT1, AMPKA, HIF1A*, and *GLUT1*) and inflammatory (*NFKB1, NFKB1A, IFNG*, and *SOCS1*) gene expression, and flow cytometry-based assessment of functional markers in NK cells from severe COVID-19 patients (n=15) and the control group (n=10), and their association with clinical outcomes. Severe COVID-19 patients exhibited elevated IFNγ, Granzyme B, and KIR2DL1 expression in NK cells compared to controls (*P < 0.005*), while LAMP1 was unchanged (*P > 0.05).* NK cells from deceased patients exhibited significantly lower expression levels of LAMP1 and Granzyme B (*P < 0.05*). Patients hospitalized >7 days presented lower Granzyme-B+ NK cells (*P < 0.05*). NK cells from severe COVID-19 patients showed downregulation of *HIF1A* and *GLUT1*, and upregulation of *NFKB1* (*P < 0.05*). *HIF1A* and *GLUT1* expression were elevated in patients with >7 days of hospitalization (*P < 0.05*). SIRT1 expression was higher in patients requiring intubation (*P < 0.05*). *SIRT1, HIF1A*, and *GLUT1* were upregulated in deceased patients (*P < 0.05*). In conclusion, we demonstrate that NK cells from patients with severe COVID-19 exhibit increased functional markers and dysregulated metabolic gene expression associated with clinical outcomes.

## Introduction

Coronavirus disease 2019 (COVID-19) is a viral infection caused by severe acute respiratory syndrome coronavirus 2 (SARS-CoV-2). Clinical manifestations range from asymptomatic or mild symptoms to severe cases requiring hospitalization and ventilatory support (Chilamakuri and Agarwal, 2021; Nasrollahi et al., 2023). It is now well established that the host immune response plays a major role in determining the severity of COVID-19. An excessive or dysregulated immune response can exacerbate inflammation and lead to extensive lung tissue damage (Björkström et al., 2022). Therefore, the evaluation of inflammatory markers, such as IL-6 and IL-8 concentrations, is now considered a prognostic indicator of disease severity in COVID-19 patients (Li et al., 2021; D’Rozario et al., 2023).

In the antiviral immune response, Natural Killer (NK) cells play a critical role in viral clearance. NK cells are innate lymphocytes that comprise approximately 1–5% of circulating peripheral blood leukocytes and serve as a first line of immune defense due to their ability to eliminate target cells without priming. Their main effector functions include cytotoxicity against tumor cells and virus-infected cells (Cichocki et al., 2019; Stokic-Trtica et al., 2020). During the COVID-19 pandemic, several studies investigated NK cell phenotypes and functions in relation to clinical outcomes (Lombardi et al., 2020; Mazzoni et al., 2020; Song et al., 2020; Yang et al., 2020; Zheng et al., 2020; Bergantini et al., 2021; Ni et al., 2021; Varchetta et al., 2021). However, their role in SARS-CoV-2 infection remains controversial. Some studies suggest that NK cells exhibit a hypofunctional phenotype, potentially impairing viral clearance and worsening disease prognosis (Li et al., 2020). In contrast, other reports have observed increased NK cell activity, suggesting a possible contribution to the excessive inflammation observed in severe COVID-19 (Malengier-Devlies et al., 2022).

Recent research has also highlighted the critical role of cellular metabolism in regulating the function of immune cells (Assmann et al., 2017; Ayres, 2020; Alqarni et al., 2021; Gassen et al., 2021; Aslan et al., 2023). In NK cells, activation is accompanied by distinct metabolic reprogramming to meet the elevated energy demands associated with their effector functions. Specifically, glycolytic and lipid metabolism pathways have been shown to play key roles in supporting the cytotoxic and cytokine-producing functions of NK cells (Osuna-Espinoza and Rosas-Taraco, 2023). However, the nature and magnitude of metabolic changes in NK cells during SARS-CoV-2 infection remain to be fully characterized.

A common approach to studying metabolism involves the analysis of genes that regulate specific metabolic pathways, often referred to as “master regulators of metabolism.” These include SIRT1, AMPK, and HIF-1α, which regulate distinct metabolic pathways in response to nutrient availability. The expression of these sensors provides insight into the dominant metabolic program operating within the cell. Beyond their role in metabolic regulation, these molecules also exhibit context-specific roles in immune and inflammatory responses. SIRT1 (a NAD^+^-dependent deacetylase) regulates several physiological processes, including metabolism and inflammation (Alqarni et al., 2021; Bordoni et al., 2021), and can activate AMP-activated protein kinase (AMPK), which is involved in autophagy and cellular homeostasis (Harmel et al., 2014; Blagih et al., 2015; Day et al., 2017; Herzig and Shaw, 2018). Both sensors promote lipid metabolism and are generally associated with anti-inflammatory responses. However, in the context of certain viral infections, including MERS-CoV and SARS-CoV-2, activation of SIRT1 and AMPK has been linked to enhanced viral replication. Conversely, HIF-1α (hypoxia-inducible factor 1-alpha), when stabilized and translocated to the nucleus, promotes the transcription of genes related to glycolysis, pro-inflammatory responses, and viral replication (Codo et al., 2020; Bao and Wong, 2021; Arias et al., 2023). Although the individual roles of these metabolic regulators have been widely described, their specific contribution to the pathogenesis of severe COVID-19 remains incompletely understood.

In this context, the analysis of metabolic sensors in NK cells from patients with severe COVID-19 may offer new insights into the relationship between cellular metabolism and cell function during severe SARS-CoV-2 infection. Understanding these mechanisms may inform the development of targeted interventions to enhance antiviral immunity, prevent immune dysfunction, and guide the development of novel therapeutic strategies. In the present study, we investigated the activation, metabolic, and inflammatory status of peripheral blood NK cells in patients with severe COVID-19, as well as their association with clinical outcomes.

## Materials and methods

### Study population and sample collection

The study included blood samples from 15 patients (both male and female), aged 23 to 85 years, who were admitted to the Hospital Universitario “Dr. José Eleuterio González” between July 2022 and February 2023 with a diagnosis of severe COVID-19, as defined by the World Health Organization (WHO) criteria. COVID-19 diagnosis was confirmed for each patient using real-time reverse transcriptase polymerase chain reaction (RT-qPCR).

All patients included in this study were hospitalized within 48–72 hours of symptom onset, 10 mL of blood were collected from each patient within the first 24 hours following hospital admission (day 0), and on days 3 and 10 of hospitalization, using a vacuum collection system containing ethylenediaminetetraacetic acid (EDTA) as an anticoagulant (**Supplementary Figure S1**). For comparison, a control group of 10 healthy participants was also included. Blood samples from the control group were collected between August 2022 and November 2023 at the Department of Immunology, School of Medicine, UANL.

### NK cell isolation

Highly pure NK cells were isolated from peripheral blood mononuclear cells (PBMCs) using negative selection with magnetic beads (NK Cell Isolation Kit, Miltenyi Biotec), according to the manufacturer’s protocol. Briefly, PBMCs were obtained by density gradient centrifugation using Ficoll-Paque™ Plus (Cytiva) and resuspended in buffer before incubation with an antibody cocktail targeting non-NK cells for 5 minutes at 4°C. Subsequently, microbeads were added, gently mixed, and incubated for 10 minutes at 4°C. The labeled cells were retained on MS columns (Miltenyi Biotec) placed in a magnetic field, while the unlabeled NK cells passed through and were collected.

Post-isolation, NK cell purity and viability were assessed by flow cytometry using fluorescently conjugated antibodies against CD3 and CD56/CD16, with NK cells defined as CD3^-^CD56^+^/CD16^+^. Viability was evaluated using 7-aminoactinomycin D (7-AAD) staining to detect cells with compromised membranes. Isolated NK cells consistently exhibited >90% purity and >95% viability, confirming the suitability of the cells for downstream gene expression analysis.

### NK immunophenotype

The expression of functional markers LAMP1 (CD107a), IFNγ, Granzyme B, and KIR2DL1 was assessed on NK cells using conventional flow cytometry in total blood. Briefly, 100 μL of anticoagulated blood was stained with fluorescence-labeled antibodies: anti-CD3, anti-CD56/CD16, anti-CD107a, anti-KIR2DL1, anti-IFNγ, and anti-Granzyme B (**Supplementary Table S1**), for 30 min at 4°C and light-protected. A permeabilization step was incorporated before staining for IFNγ and Granzyme B evaluation using the Cytofix/Cytoperm Fixation/Permeabilization Kit (BD Biosciences). Specific fluorescence was determined by using fluorescence minus one (FMO) control. After staining, at least 5,000 events were acquired in a BD LSRFortessa flow cytometer and analyzed with FACS Diva software v8.0 (**Supplementary Figure S2**).

### Gene expression

Metabolic and inflammatory gene expression was analyzed in previously isolated NK cells from the peripheral venous blood of severe COVID-19 patients and healthy controls. Total RNA was isolated using the Trizol reagent (Invitrogen) according to the manufacturer’s instructions. Briefly, NK cells isolated by magnetic column separation were pelleted and resuspended in 1 mL of TRIzol reagent (Cat. #15596018). The suspension was homogenized by vortexing, followed by the addition of 200 µL of chloroform. After mixing, samples were incubated on ice for 10 minutes. Following centrifugation, the aqueous phase was transferred, and 500 µL of isopropanol was added. The mixture was homogenized and incubated to precipitate RNA. The supernatant was discarded after centrifugation, and the RNA pellet was washed with 1 mL of 75% ethanol. The pellet was then vortexed, centrifuged, and the supernatant was removed. Finally, the RNA pellet was resuspended in DEPC-treated water, and RNA concentration and purity were assessed using a NanoDrop spectrophotometer.

The cDNA was reverse transcribed from 100 ng of RNA using the iScript cDNA Synthesis Kit (Bio-Rad). Real-time PCR was performed using 100 ng of cDNA and iQ SYBR Green Supermix (Bio-Rad) on a CFX96 thermal cycler (Biorad). Specific primers and/or TaqMan probes (Thermo Fisher Scientific) used in RT-PCR are listed in **Supplementary Table S2**. Gene expression analysis was performed using the Livak method (2^-ΔΔCt^) (Livak and Schmittgen, 2001). *GAPDH* was used as the reference gene for normalization based on a review of the relevant literature. Its selection was based on a review of the relevant literature (Feng et al., 2017; Park et al., 2021; Wang et al., 2025), followed by validation of its expression stability. Gene expression analysis was performed using the Livak method (2^−ΔΔCt^).

### Statistical analysis

The normality of the data distribution was evaluated using the Shapiro–Wilk test. Comparisons between groups were performed using Student’s t-test or the Mann-Whitney U test for parametric or non-parametric data, respectively. Pearson’s correlation test was performed for parametric variables, and Spearman’s correlation for non-parametric variables. Statistical analysis was performed in GraphPad Prism v5.0, and a *P-*value <0.05 was considered statistically significant.

## Results

### Demographic and clinical characteristics of participants

The mean age was 64.7 ± 18.1 years for patients with severe COVID-19 and 65.8 ± 9.2 years for the control group, with no significant difference (*P = 0.661*). Female patients represented 53.3% of the severe COVID-19 group, a proportion similar to that observed in the control group (*P = 0.519*). The most prevalent comorbidities in the severe COVID-19 cohort were type 2 diabetes and hypertension, which were present in 60% of patients. The clinical characteristics of patients with severe COVID-19 were stratified based on the hospitalization length, the need for invasive ventilatory support, and clinical outcome (discharge status). The median length of hospital stay was 8 days (1–38 days). Among the patients, 60% required non-invasive ventilatory support (nasal cannula), while 40% required invasive ventilatory support (tracheal intubation). In the clinical outcome, 60% of patients were discharged following medical improvement. The clinical characteristics of the severe COVID-19 patients are summarized in **Supplementary Table S3**.

### Granzyme B and LAMP1 expression in NK cells are associated with hospital stay and clinical outcomes in patients with severe COVID-19

**Figure 1:**
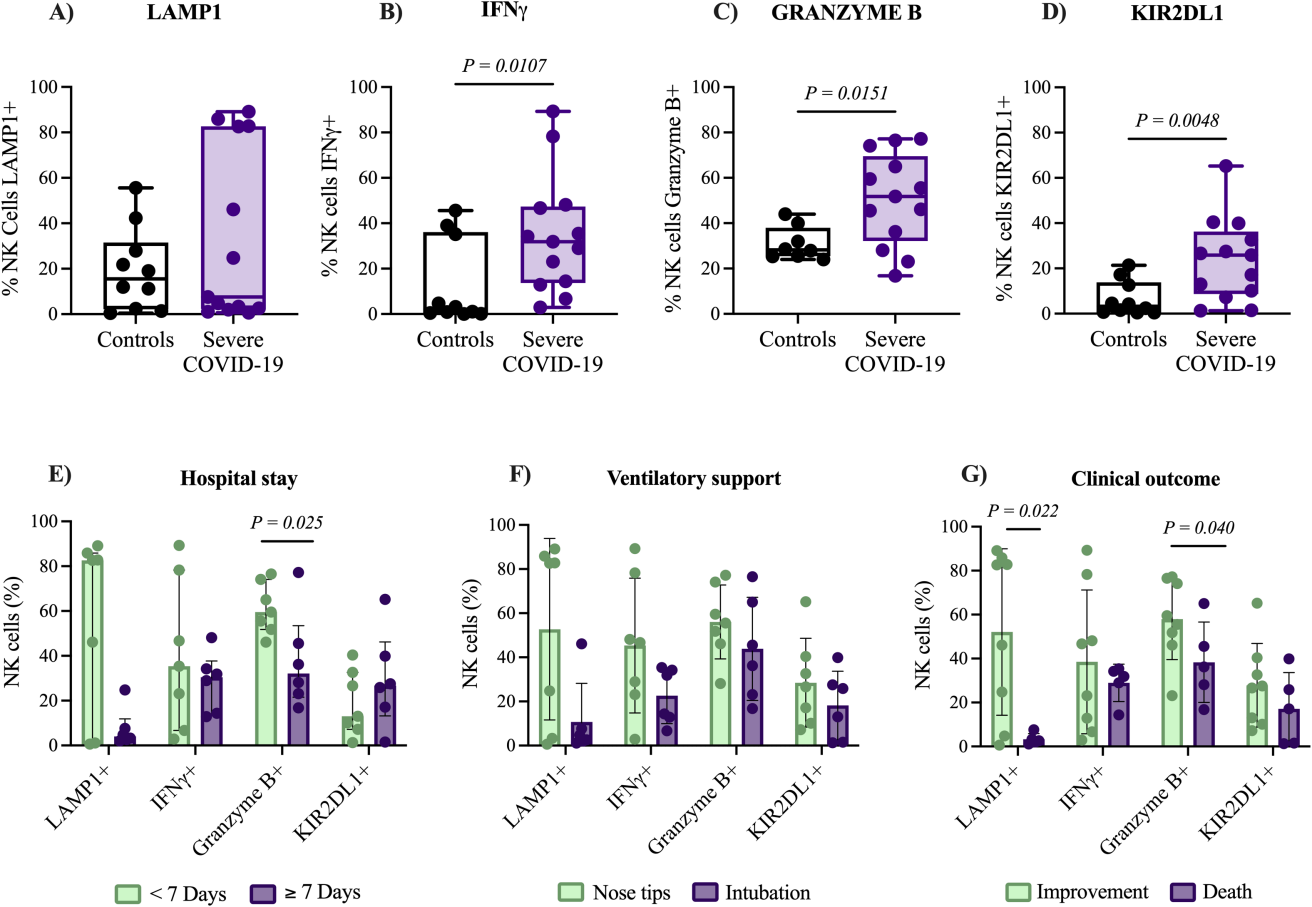
Expression of functional markers in NK cells and clinical outcomes of patients with severe COVID-19. Comparison of functional marker expression in NK cells using flow cytometry analysis between patients with severe COVID-19 (n= 15) and control group (n= 10). Markers analyzed include LAMP1 **(A)**, IFN-γ **(B)**, Granzyme B **(C)**, and KIR2DL1 **(D)**. The expression levels of these markers were further examined in relation to **(E)** duration of hospital stay (short vs. long), **(F)** requirement for ventilatory support (non-invasive vs. invasive), and **(G)** clinical outcomes (discharge vs. death). Data distribution was evaluated using the Shapiro-Wilk test, and group comparisons of medians were performed with the Mann–Whitney U test. The P-values are shown in the figure.

Upon activation, NK cells initiate a cytotoxic response that includes the secretion of cytokines and the release of cytotoxic granules. To assess the functional status of NK cells in this study, we evaluated the expression of classical functional markers, including LAMP1, IFN-γ, and Granzyme B, as well as the inhibitory receptor KIR2DL1. Our results show that patients with severe COVID-19 exhibit significantly higher percentage of NK cells positive to the functional markers IFN-γ (31.9% [2.9-89.3]) compared to the control group (2.15% [0-45.6]), *P = 0.010*; and Granzyme B (51.8% [16.8-77.2]) compared to the control group (28.2% [24-44]), *P = 0.015*. As well as the inhibitory receptor KIR2DL1 (25.9% [1.3-65.2]) compared to the control group (3.30% [0.5-21.4]), *P = 0.004;* at hospital admission, see **Figure 1**. Alterations in the expression of activation and inhibition markers on NK cells observed at hospital admission remained stable throughout the hospital’s length of stay. There were no significant differences in expression levels across subsequent blood samples (days 3 and 10 of the hospital stay), as shown in **Supplementary Figure S3**.

Consequently, we investigated whether the expression of activation and inhibition markers in NK cells from patients with severe COVID-19 was associated with clinical outcomes. We observed that patients hospitalized for more than 7 days exhibited a significantly lower percentage of NK cells Granzyme B+ (32.1% [16.8-77.2]), compared to those hospitalized for less than 7 days (59.5% [46.1-76.5]), *P = 0.025*. Under these associations, the length of hospital stay was significantly correlated with the expression of LAMP1 (*P = 0.044, r = -0.493*) and Granzyme B (*P = 0.020, r = -0.579*), as shown in **Supplementary Figure S4**. Additionally, deceased individuals exhibited significantly lower percentage of NK cells expressing Granzyme B (36.2% [16.8-65]) compared to patients who improved (57.5% [23.1-77.2]), *P = 0.040*; also deceased patients showed lower percentage of NK cells expressing the degranulation marker LAMP1 (2.6% [1.1-7.6]) compared to patients who improved (64.35% [0.6-89.1]), *P = 0.022*. In contrast, no associations were found between the expression of IFN-γ or KIR2DL1 and clinical outcomes (*P = 0.471 and P = 0.142*, respectively), **Figures 1E-G**. These findings indicate an activated NK cell phenotype in patients with severe COVID-19, and the elevated levels of LAMP1 and Granzyme B are associated with favorable clinical outcomes.

### Downregulation of HIF1A and GLUT1 in NK cells of patients with severe COVID-19

**Figure 2:**
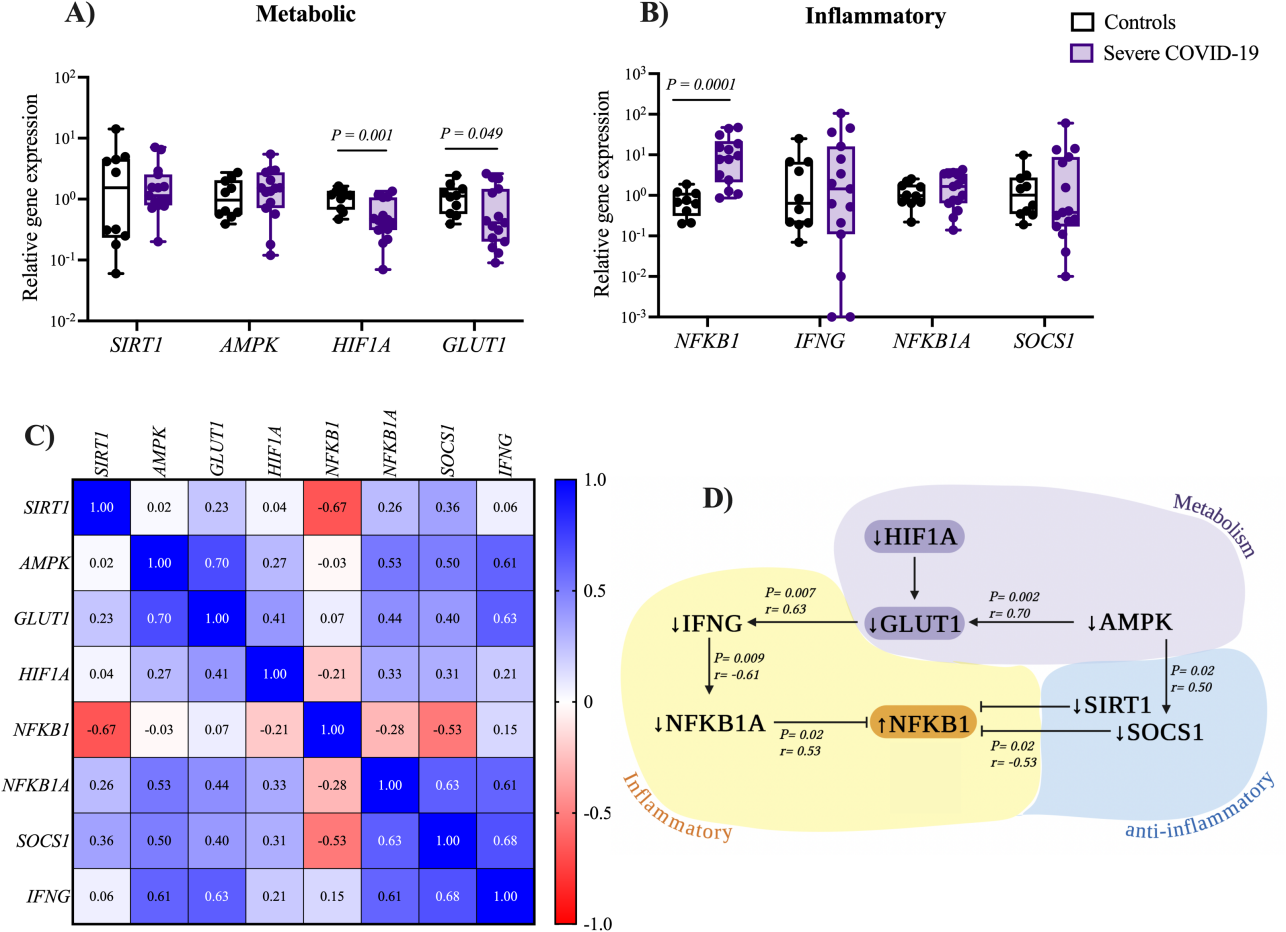
Expression levels of metabolic and inflammatory genes in NK cells from patients with severe COVID-19. Relative expression levels of **(A)** metabolic and **(B)** inflammatory-related genes in NK cells were compared between patients with severe COVID-19 (n= 15) and the control group (n= 10). Data distribution was assessed using the Shapiro-Wilk test, and differences between groups were analyzed using the Mann–Whitney U test; P-values are shown in the figure. **(C)** Correlation matrix heatmap showing associations between metabolic and inflammatory gene expression in NK cells from patients with severe COVID-19. Each heatmap cell is colored according to the P value and labeled with the corresponding Spearman correlation coefficient (R value). **(D)** Schematic representation of significant correlations identified between metabolic and inflammatory gene expression in NK cells from patients with severe COVID-19.

During cell activation, metabolic changes occur to meet the energy demands of different cellular functions. In this direction, we evaluated metabolic alterations in genes recognized as a master regulator of cellular metabolism in isolated NK cells from patients with severe COVID-19. No significant differences were observed in the expression of *SIRT1* on NK cells from severe COVID-19 patients (1.14 [0.2-7.1]) compared to the control group (1.53 [0.06-14.1]), *P= 0.382*. Similar results were found for *AMPK* expression in NK cells from severe COVID-19 patients (1.51 [0.1-5.4]) compared with the control group (0.96 [0.39-2.74]), *P = 0.165*; as shown in **Figure 2A**. In contrast, *HIF1A* expression was 2.6-fold lower in NK cells of patients with severe COVID-19 (0.45 [0.07-1.35]) than in the control group (1.17 [0.47-1.63]), *P = 0.001*. Similarly, results were observed in *GLUT1* expression, which was 2.7-fold lower in patients with severe COVID-19 (0.41 [0.09-2.62]) compared to controls (1.12 [0.39-2.45]), *P = 0.049*. *HIF1A* and *GLUT1* are key regulators of anaerobic glycolysis. Therefore, their downregulation suggests a shift toward reduced glycolysis in NK cells from patients with severe COVID-19. *SIRT1, AMPK, HIF1A*, and *GLUT1* expression levels showed no significant differences compared to subsequent blood samples on days 3 and 10 (see **Supplementary Figure S5**). These results suggest that the expression of these metabolic genes remains stable in patients with severe COVID-19.

To investigate the potential role of inflammation-related genes in NK cells from patients with severe COVID-19, we examined the expression of *NFKB1* and *IFNG*, as shown in **Figure 2B**. We found a significant upregulation (11.3-fold higher) of *NFKB1* in severe COVID-19 patients (7.73 [0.86-47.18]) compared to the control group (0.68 [0.20-1.89]), *P = 0.0001*. On the other hand, no significant differences were found for *IFNG* expression between severe COVID-19 patients (1.44 [0-105.4]) and the control group (0.64 [0.07-24.93), *P = 0.408*. The expression levels of the anti-inflammatory gene *NFKB1A*, an inhibitor of the pro-inflammatory transcription factor NFKB1, were higher in severe COVID-19 patients (1.65 [0.14-4.29]) compared to the control group (0.98 [0.22-2.55]). However, the difference was not statistically significant (*P = 0.202*). Similar results were observed for *SOCS1*, a key suppressor of cytokine signaling, in severe COVID-19 patients (0.38 [0.01-60.1]) compared to the control group (1.01 [0.19-9.78]), *P = 0.269*, as shown in **Figure 2B**. These results indicate that NK cells from patients with severe COVID-19 exhibit an inflammatory state. Comparison of inflammation-related gene expression with subsequent blood samples collected on days 3 and 10 revealed a significant downregulation of both *NFKB1 (P = 0.026)* and its inhibitory regulator *NFKB1A* (*P = 0.015*) at day 10 of hospitalization, indicating a potential reduction in the inflammatory response throughout hospitalization. In contrast, the expression levels of *SOCS1* and *IFNG* remained stable throughout the hospital stay in patients with severe COVID-19 (see **Supplementary Figure S6**).

We conducted a correlation analysis to identify potential links between metabolic and inflammatory genes (**Figure 2C**). Our study revealed a positive correlation between *AMPK* expression and both *GLUT1* (*P = 0.002, R = 0.70*) and *SOCS1* (*P < 0.029, R = 0.50*). A positive correlation was also observed between GLUT1 and *IFNG* expression (*P < 0.007, R = 0.63*). **Figure 2D** illustrates the most significant correlations between metabolic and inflammatory genes.

### Overexpression of metabolic genes in NK cells as indicators of adverse outcomes in severe COVID-19 patients

**Figure 3:**
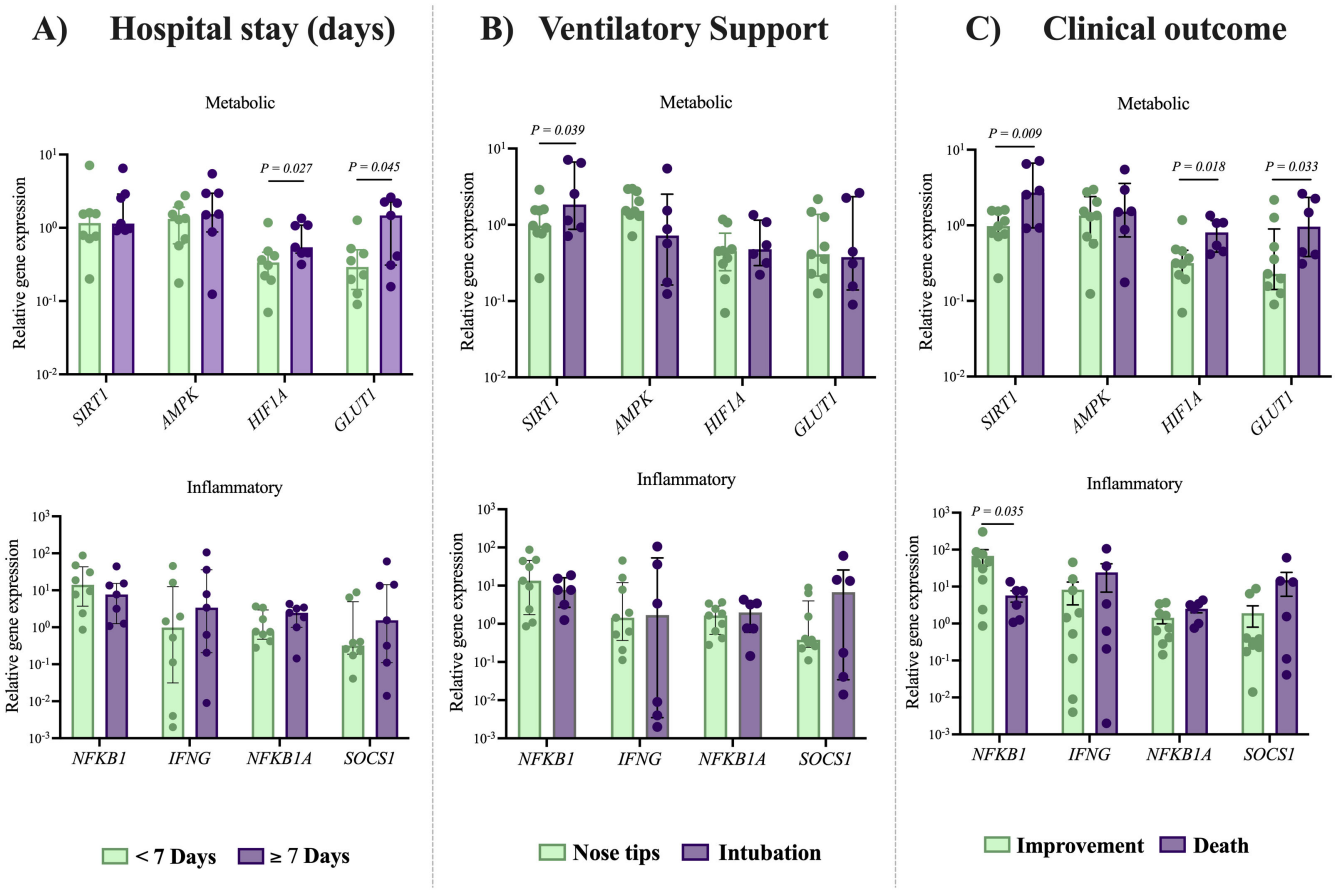
Expression of metabolic and inflammation-related genes in NK cells according to clinical outcomes in patients with severe COVID-19. Relative expression levels of metabolic and inflammation-related genes were analyzed in NK cells from patients with severe COVID-19 (n= 15), with comparisons made according to key clinical parameters: **(A)** duration of hospital stay (short <7 days vs. long ≥7 days), **(B)** type of ventilatory support required (non-invasive vs. invasive), and **(C)** clinical outcome (discharge vs. death). Expression levels were obtained via RT-qPCR and normalized to GAPDH using the Livak method. For reference, expression levels in NK cell from a control group (n= 10) were assessed. Data distribution was evaluated using Shapiro Wilk test and group comparisons were performed using the Mann–Whitney U test; P-values are shown in the figure.

After analyzing the expression of metabolic and inflammatory genes in NK cells from patients with severe COVID-19, we evaluated whether their expression was associated with disease severity based on three clinical characteristics: length of hospital stay, need for invasive or non-invasive ventilatory support, and patient outcome (improvement or death). We observed significantly higher expression of *HIF1A* in patients hospitalized for 7 days or longer (1.6-fold higher, 0.54 [0.31-1.34]) compared to those hospitalized for less than 7 days (0.33 [0.07-1.17]), *P = 0.027*. Similar results were observed for *GLUT1;* patients with more extended hospital stays exhibited higher expression (5-fold higher, 1.47 [0.15-2.62]) compared to those with shorter hospital stays (0.29 [0.09-1.26]), *P = 0.045*. No significant differences were observed between the groups in the expression of inflammatory-related genes (see **Figure 3A**).

Regarding ventilatory support, we found that intubated patients exhibited increased expression of the metabolic gene *SIRT1* (2.3-fold higher, 1.84 [0.7-7.1]) in NK cells from severe COVID-19 patients, compared to those receiving non-invasive ventilatory support (0.97 [0.2-2.8]), *P = 0.039*, see **Figure 3B**. Finally, we also compared the expression of metabolic and inflammatory genes in NK cells from patients with severe COVID-19 who recovered and those who died. A significant downregulation of the inflammatory gene *NFKB1* (3.4-fold lower) in fatal cases (5.45 [1.08-13.45]) compared with recovered ones (18.7 [0.8-87.4]), *P = 0.035* was shown. These findings support the relation between an insufficient inflammatory response and poor clinical outcomes in patients with severe COVID-19.

**Figure 4:**
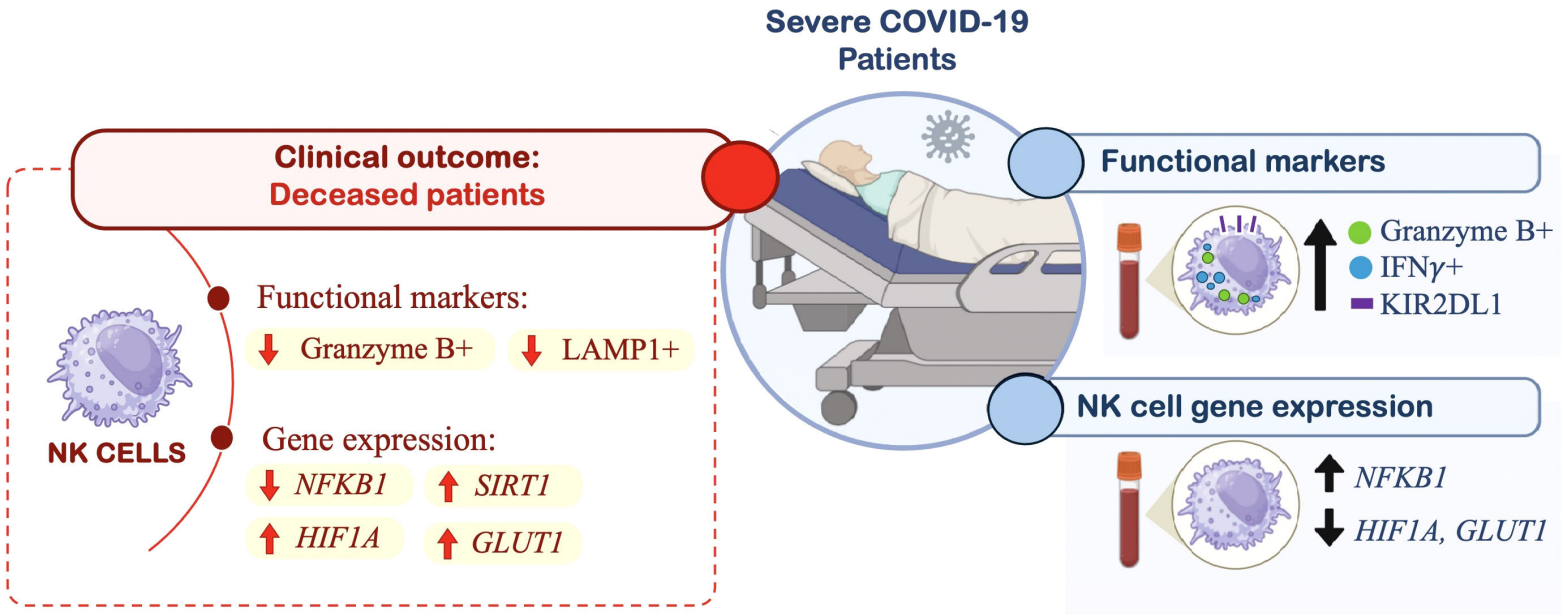
Graphical abstract of study results. This schematic summarizes key immunophenotypic and gene expression changes observed in peripheral blood NK cells from patients with severe COVID-19 (n= 15), compared to a control group (n= 10), and their association with clinical outcomes (deceased patients). In severe COVID-19 patients, flow cytometry analyses revealed increased expression of Granzyme B, IFN-γ, and KIR2DL1 in NK cells. Gene expression profiling using RT-qPCR showed upregulation of the inflammatory gene NFKB1, along with downregulation of metabolic genes HIF1A and GLUT1 in NK cells from severe cases. Further subgroup analysis based on clinical outcome (discharge vs. death) revealed that deceased patients exhibited lower expression of Granzyme B and LAMP1, but higher expression of HIF1A, GLUT1, and SIRT1.

Among the metabolic genes analyzed, we observed an upregulation of *SIRT1* (2.7-fold higher; 2.7 [0.9-7.1]), *P = 0.009*, *HIF1A* (2.5-fold higher; 0.80 [0.4-1.3]), *P = 0.018*, and *GLUT1* (4.2-fold higher, 0.95 [0.3-2.6]), *P = 0.033* in deceased patients, compared to recovered patients 0.97 [0.2-1.6], 0.31 [0.07-1.17] and 0.22 [0.09-2.17], respectively), see **Figure 3**. These findings highlight the contribution of cellular metabolic processes in modulating the immune response against SARS-CoV-2 infection (**Figure 4**).

## Discussion

In viral infections, such as COVID-19, NK cells play an essential role in viral clearance and pathogenesis. The functional status of NK cells during SARS-CoV-2 infection remains a subject of active investigation. Some studies report a hypofunctional phenotype linked to poor viral control and clinical outcomes (Bobcakova et al., 2021), while more recent findings suggest that increased NK cell activity contributes to the inflammatory response in COVID-19. Given these contrasting observations, the present study aims to assess the functional status of circulating NK cells in patients with severe COVID-19. Additionally, given the emerging role of cellular metabolism in immune regulation, we evaluated the expression of metabolic and inflammatory genes and their association with NK cell function and clinical outcomes of severe COVID-19 patients.

In this study, we evaluated three classical functional markers and an inhibitory receptor of NK cells: LAMP1 (CD107a), a degranulation marker; IFN-γ, a proinflammatory cytokine secreted upon activation; Granzyme B, a cytotoxic granule; and KIR2DL1, a classical inhibitory receptor. Our results demonstrate an overexpression of the functional markers IFN-γ and Granzyme B, as well as the inhibitory receptor KIR2DL1, on peripheral blood NK cells from severe COVID-19 patients, compared to uninfected controls.

The increased expression of these markers at the protein level is indicative of a functionally active state of NK cells. In contrast to activating receptors, whose expression alone is not necessarily indicative of cellular activation, these functional markers are upregulated after NK cell activation and correlate directly with their effector functions. Consequently, their expression represents a more reliable indicator of NK cell functional status. These findings suggest that circulating NK cells in severe COVID-19 exhibit a functional phenotype, marked by elevated cytotoxic mediators characteristic of NK cell function.

Existing data on IFN-γ expression in NK cells during SARS-CoV-2 infection remain inconsistent. While some studies have reported no significant change in IFN-γ expression (Mazzoni et al., 2020; Osman et al., 2020), others have observed a decrease (Zheng et al., 2020; Krämer et al., 2021). In contrast, our findings are consistent with recent work by Bert M. et al (Malengier-Devlies et al., 2022), who similarly reported upregulation of both IFN-γ and LAMP1 in peripheral blood NK cells from patients with severe COVID-19.

Several studies have reported elevated serum Granzyme B levels in COVID-19 patients, reflecting the activation of cytotoxic lymphocytes (Li et al., 2020; Bergantini et al., 2021). In line with these findings, our study demonstrated a significant increase in Granzyme B expression in NK cells from patients with severe COVID-19. This observation is consistent with the results of Maucourant et al (Maucourant et al., 2020), who also reported elevated expression of this cytotoxic mediator in patients with severe disease. In addition to functional markers, we evaluated the expression of KIR2DL1, an inhibitory receptor belonging to the KIR family, and found it overexpressed on NK cells from patients with severe COVID-19. Similar findings were reported by other authors (Demaria et al., 2020; Saresella et al., 2021), highlighting the role of activating and inhibitory receptor co-expression in regulating NK cell function. The discrepancies observed in NK cell functional marker expression across different studies may be primarily attributable to variations in methodology. Decisively, the lack of stratification by disease severity in some reports may limit the detection of critical immunological differences, as the intensity of the immune response is closely linked to the clinical stage of COVID-19.

Importantly, our findings highlight a complex aspect of NK cell responses in COVID-19. While patients with severe disease exhibited elevated activation markers such as Granzyme B and LAMP1 at hospital admission, reduced expression of these markers was associated with worse clinical outcomes, including prolonged hospitalization and mortality. These results suggest that although NK cells are activated in response to severe infection, the preservation of their cytotoxic function may be critical for effective immune defense and favorable recovery.

A possible explanation for these observations is that NK cells from patients with poor outcomes are intrinsically less responsive to activation signals. An alternative hypothesis is that NK cells may initially become activated but subsequently undergo functional exhaustion or dysregulation as the disease progresses. However, in our cohort, we observed no significant changes in the expression of activation or inhibitory markers between admission and subsequent time points (days 3 and 10), suggesting a pattern of early functional impairment rather than progressive exhaustion. While these findings provide valuable insights, they remain inconclusive and underscore the need for further studies that include patients with a broader range of disease severity, including those with mild or moderate illness, would offer a more complete understanding of NK cell regulation during SARS-CoV-2 infection and its impact on disease outcomes.

Additionally, we assessed the expression of inflammation-related genes in NK cells and observed an upregulation of *NFKB1*. These findings are consistent with the inflammatory state previously reported in patients with severe COVID-19 (Wilk et al., 2020). Although elevated IFN-γ protein levels were detected by flow cytometry in NK cells from patients with severe COVID-19, *IFNG* transcript levels did not differ significantly between groups.

Studies have demonstrated that only ~40% of protein variability is explained by mRNA levels, with the remainder influenced by translation efficiency and protein stability (De Sousa Abreu et al., 2009). Viral infections, including SARS-CoV-2, can enhance protein synthesis without altering the abundance of mRNA. IFN-γ production, for instance, can increase independently of *IFNG* transcription following viral stimulation (Ben-Asouli et al., 2000). In NK cells, high-dose IL-15 activates translation via mTORC1, thereby enhancing the phosphorylation of initiation factors and increasing the output of IFN-γ protein. Co-stimulation with IL-12, IL-18, or activating receptors further amplifies this effect, promoting translation independently of transcript levels (Keppel et al., 2015; Cimpean et al., 2023). The elevated levels of IFN-γ protein observed in this study likely reflects translational regulation and highlight the value of integrating transcriptomic and proteomic data to characterize immune responses in severe viral infections fully.

A key metabolic regulator evaluated in this study is SIRT1, a NAD^+^-dependent deacetylase that modulates antiviral immune responses. While prior *in vitro* studies have suggested conflicting roles for SIRT1 in SARS-CoV-2 infection, ranging from antiviral activity to supporting viral replication, clinical evidence remains limited and inconclusive (Walter et al., 2022; Zhang et al., 2022). Our study, to the best of our knowledge, is the first to evaluate *SIRT1* expression in NK cells isolated from patients with severe COVID-19. Although no statistically significant differences in *SIRT1* expression were observed compared to the control group, we identified a substantial relation between *SIRT1* expression in NK cells and clinical outcomes. Notably, patients who required invasive mechanical ventilation and those with fatal outcomes exhibited elevated levels of *SIRT1* expression in NK cells. These findings suggest a potentially adverse role of *SIRT1* in the context of severe COVID-19. However, further studies are required to elucidate the precise role of *SIRT1* in COVID-19.

*SIRT1* has been described to regulate downstream targets, such as *AMPK*, another key metabolic sensor. In our cohort, the expression of *AMPK* in NK cells did not differ significantly between COVID-19 patients and the control group. Furthermore, *AMPK* expression was not associated with clinical outcomes in these patients. To date, no studies have specifically evaluated *AMPK* expression in NK cells. Existing research has primarily examined the role of *AMPK* in SARS-CoV-2 infection using *in vitro* models with established cell lines; however, the findings reported to date remain contradictory. Jang et al. demonstrated a reduced replication of SARS-CoV-2 in cells lacking (Jang et al., 2023). In contrast, Gassen et al. reported that SARS-CoV-2 infection induces the downregulation of active forms of *AMPK* and AMPK-related signaling genes (Gassen et al., 2021). These observations have prompted the investigation of AMPK-activating drugs as potential therapeutic agents for COVID-19 (Parthasarathy et al., 2023). Our study specifically evaluated *AMPK* expression in peripheral blood NK cells. Although it is now recognized that SARS-CoV-2 can infect NK cells, this population is not considered a primary or preferential target of the virus; however, this may nonetheless directly influence the expression of this gene (Matveeva et al., 2022).

Among the metabolic genes analyzed, *HIF1A* has been the most extensively investigated in the context of SARS-CoV-2 infection. *HIF1A* activation led to metabolic reprogramming that favors glycolysis and promotes inflammatory responses via NF-κB signaling pathways. Although limited, current studies report an upregulation of *HIF1A* expression in PBMCs from patients with COVID-19 (Codo et al., 2020; Tian et al., 2021; Karabulut Uzunçakmak et al., 2022), and an association of *HIF1A* expression with viral replication (Duan et al., 2021). This is primarily attributed to the increased metabolic demands of SARS-CoV-2-infected cells required for viral replication, suggesting that *HIF1A* may facilitate viral replication. In contrast to previous studies that report HIF1A upregulation in PBMCs, we observed significantly reduced *HIF1A* and *GLUT1* expression in NK cells from patients with severe COVID-19.

Although our initial results demonstrated significant downregulation of *HIF1A* and *GLUT1* in NK cells from patients with severe COVID-19 compared to healthy controls, further stratification by clinical outcome revealed that patients with fatal outcomes exhibited elevated expression of *SIRT1*, *HIF1A*, and *GLUT1*. This finding aligns with previous reports linking *HIF1A* expression to poorer clinical outcomes (Codo et al., 2020; Tian et al., 2021; Karabulut Uzunçakmak et al., 2022). The apparent discrepancy may reflect the dynamic, context-dependent nature of NK cell metabolic responses during SARS-CoV-2 infection. The upregulation of these metabolic genes in fatal cases suggests a dysregulated shift toward glycolytic and lipid metabolism in NK cells, which may contribute to disease progression. These observations are supported by recent studies that associate these metabolic pathways with disease severity in patients with severe COVID-19 (Chung et al., 2024). Collectively, these results reveal a heterogeneous metabolic profile in NK cells during severe COVID-19 and highlight the importance of clinical context when evaluating immune-metabolic biomarkers in COVID-19.

However, additional functional analyses are needed to clarify the specific impact of these metabolic genes on NK cell function and their contribution to clinical outcomes in patients. As an initial approach, we investigated the relationship between the expression of metabolic genes and key functional markers of NK cells. The lack of significant correlations between *HIF1A* and canonical NK cell effector markers (LAMP1, IFN-γ, Granzyme B, KIR2DL1) suggests that *HIF1A* may not directly regulate cytotoxic activity, but instead, exert an indirect or context-dependent effect. Based on these findings, we hypothesize that the association of *HIF1A* with clinical outcomes may reflect a broader inflammatory status, as supported by the upregulation of the inflammatory gene *NFKB1*.

To our knowledge, this is the first study to assess the expression of these metabolic genes in NK cells isolated from patients with COVID-19. Emerging approaches have proposed the use of inhibitors and activators of these metabolic sensors as potential therapeutic strategies for patients with COVID-19 (Ramdani and Bachari, 2020; Kamyshnyi et al., 2021; Parthasarathy et al., 2023). However, further research is necessary to assess the clinical significance of these sensors and their possible implications for disease severity.

Several factors may account for the discrepancies between our findings and those previously reported in the literature. Notably, most studies on SARS-CoV-2 infection have assessed the metabolic gene expression in PBMCs, whereas our research analyzed its expression specifically in isolated NK cells without stimulation. Although NK cells can be directly infected by SARS-CoV-2 and exhibit alterations in energy metabolism, current evidence suggests that the virus predominantly infects monocytes, macrophages, dendritic cells, and B cells among immune cell populations (Matveeva et al., 2022). This may represent the primary factor driving cellular metabolic alterations and modulating the expression of these metabolic regulatory genes. Other studies assessing the expression of these metabolic genes in the context of SARS-CoV-2 infection have been conducted using *in vitro* models with established cell lines. Only a limited number of studies have analyzed samples from COVID-19 patients, and among those, few have stratified the data based on disease severity, a critical determinant of the immune response. Studies have demonstrated that the severity of infection significantly influences the nature and magnitude of immune alterations (Song et al., 2020). This study specifically evaluated samples from patients with COVID-19 classified as severe; therefore, the results may not be directly comparable to those of other studies, particularly due to differences in study design and patient populations.

Limitations of this study include the relatively small sample size, which limits the statistical power to detect differences between groups, particularly in longitudinal comparisons, thereby increasing the risk of type II errors. The post-vaccination phase of the pandemic posed additional challenges in the recruitment of patients with severe illness. Although our cohort is comparable to those in previous studies of severe COVID-19 (Codo et al., 2020; Kang et al., 2020; Rha et al., 2021), larger and multicenter cohorts are required to validate and extend these findings. Valuable insights could be gained by comparing patients with mild, moderate, and severe COVID-19 to assess the potential association of metabolic alterations and disease severity. Furthermore, the analysis of publicly available datasets could enhance the identification of a prognostic signature based on the immunological markers evaluated in this study.

Another limitation is the potential impact of hospital-administered treatments. During hospitalization, patients received diverse medical interventions, including immunomodulatory therapies, which may have influenced NK cell function and gene expression profiles in subsequent samples. Despite these limitations, relatively few studies have explored the expression of metabolic regulators in NK cells during SARS-CoV-2 infection. Our findings contribute to this emerging area by identifying potential associations between metabolic gene expression in NK cells and the inflammatory response observed in severe COVID-19. These findings can be enriched with additional studies evaluating a broader panel of metabolic genes and signaling pathways, emerging evidence indicates that dysregulation of fatty acid oxidation pathways is linked to the severity and clinical prognosis of critically ill COVID-19 patients (Chung et al., 2024); Also, incorporating functional metabolic assays, such as Seahorse extracellular flux analysis, to more comprehensively characterize the metabolic programming of NK cells during viral infection.

Our results suggest a potential role of NK cell metabolism in the clinical outcomes of patients with severe COVID-19. Elucidating how metabolic sensors, such as HIF-1α, GLUT1, SIRT1, and AMPK, influence NK cell function in COVID-19 will enhance our understanding of disease pathogenesis and serve as a basis for novel therapeutic approaches in patients with COVID-19. In conclusion, we demonstrated the downregulation of *HIF1A* and *GLUT1* in NK cells from patients with severe COVID-19 and provided evidence of their association with clinical outcomes.

## Data Availability

The original contributions presented in the study are publicly available. This data can be found here https://doi.org/10.6084/m9.figshare.29848025.v1.
