# Supplementary material for: Expression of metabolic genes in NK cells is associated with clinical outcomes in patients with severe COVID-19: a brief report
Source: Front Cell Infect Microbiol. 2025 Aug 25;15:1636463. doi: 10.3389/fcimb.2025.1636463 (PMC12415030; doi:10.3389/fcimb.2025.1636463)
Supplement: Supplementary file 1 [file DataSheet1.docx]

**Supplementary Material**

**
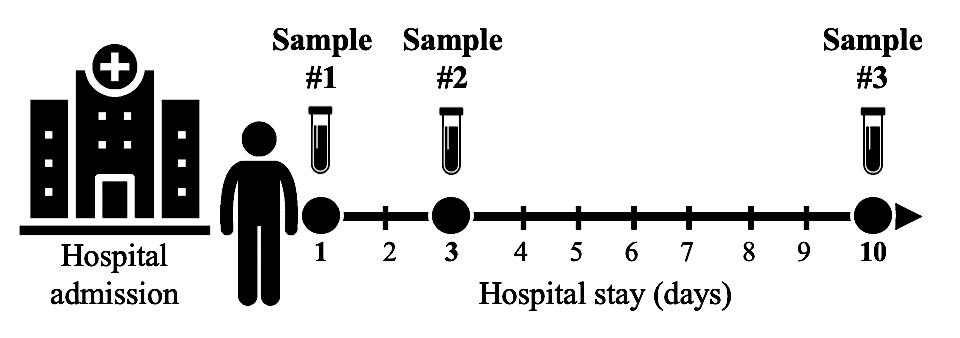
**

**Figure S1.** Schematic Representation of the Sample Collection Workflow.

**Table S1.** Antibodies Used in Flow Cytometry.

| Antibody | Clone | Fluorochrome | Company |
| --- | --- | --- | --- |
| BD simultest CD3/CD16+CD56 |  | FITC/PE | BD Bioscience |
| Mouse Anti-Human CD107a (LAMP1) | H4A3 | Pacific Blue | BD Bioscience |
| Mouse Anti-Human CD158a (KIR2DL1) | HP-3E4 | APC | BD Bioscience |
| Mouse Anti-Human IFNγ | B27 | Brilliant Violet 605 | BD Bioscience |
| Mouse Anti-Human Granzyme B | GB11 | PE | BD Bioscience |
| Mouse Anti-Human CD8 | SK1 | APC-CY7 | BD Bioscience |

**
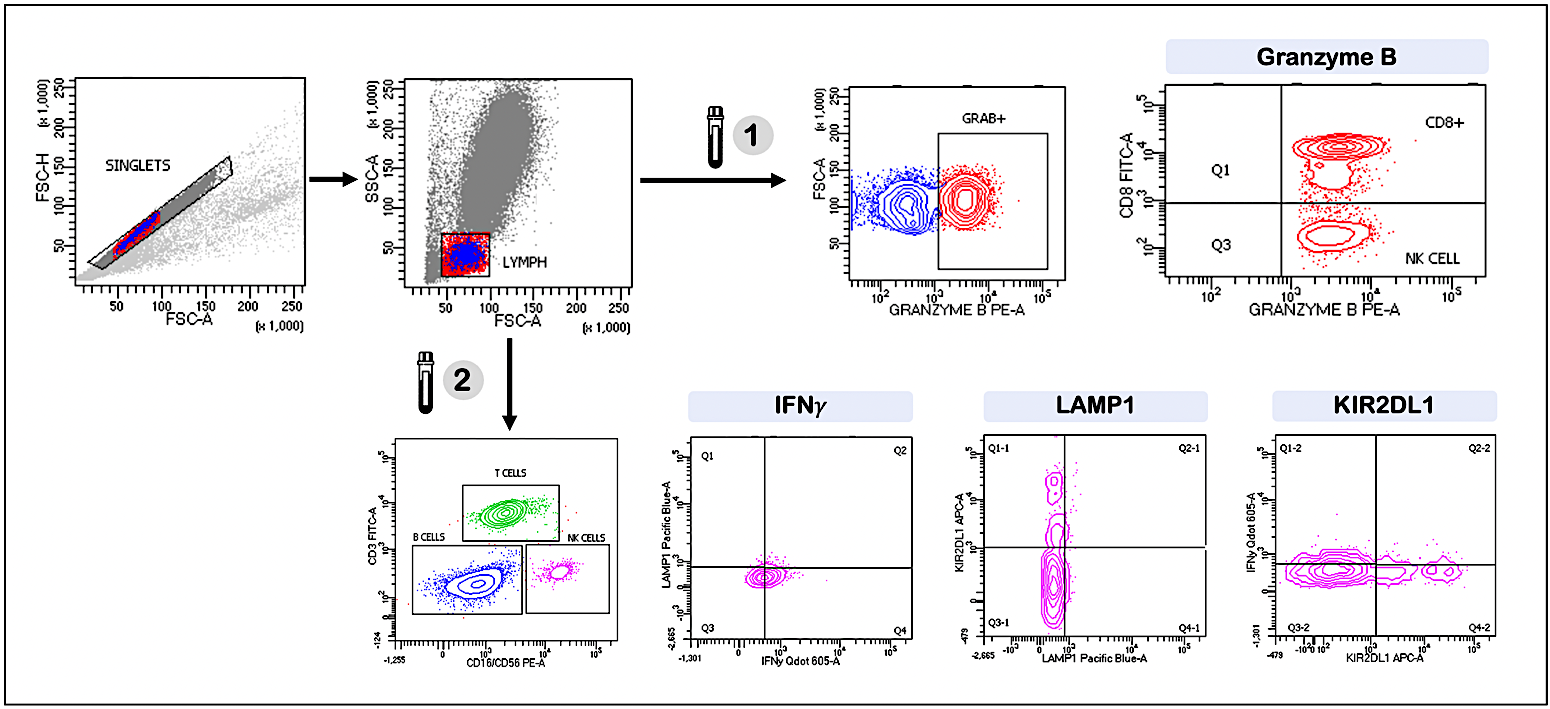
**

**Figure S2. Representative flow cytometry plots illustrating the gating strategy for the analysis of functional markers in NK cells.** Two separate staining panels (Tube A and Tube B) were used for flow cytometric analysis of NK cells from patients with severe COVID-19 and healthy controls: Tube 1 was used to assess granzyme B expression, while Tube 2 was used to evaluate IFN-γ, LAMP1, and KIR2DL1 expression.

**Table S2.** Primers And Taqman Probes Used in Real Time-PCR

| Gene | Taqman probes | Ref. |
| --- | --- | --- |
| *IFNG* | Hs00989291_m1 | (1) |
| *SOCS1* | Hs00705164_s1 | (2) |
| *NF-kB1* | Hs00765730_m1 | (3) |
| *NF-kB1A* | Hs00355671_g1 | (4) |
| Gene | **Primers** | **Ref.** |
| *AMPKA1* | F: 5’AGGAAGAATCCTGTGACAAGCAC  R: 5’CCGATCTCTGTGGAGTAGCAGT | (5,6,7,8,9) |
| *SIRT1* | F: 5’AATCCAGTCATTAAAGGTCTACAA  R: 5’TAGGACCATTACTGCCAGAGG | (10,11,12) |
| *GLUT1* | F: 5’TATGTGGAGCAACTGTGTGGT  R: 5’TCCGGCCTTTAGTCTCAGGA | (13) |
| *HIF1A* | F: 5’CAAGAACCTACTGCTAATGC  R: 5’TTATGTATGTGGGTAGGAGATG | (14,15) |
| *GAPDH* | F: 5’GACAGTCAGCCGCATCTTCT  R: 5’AAATGAGCCCCAGCCTTCTC | (16,17,18) |

**Table S3.** Clinical Characteristics of COVID-19 patients.

| **VARIABLE** | **SEVERE COVID-19**  n=15 |
| --- | --- |
| **Comorbidities** | |
| Type 2 Diabetes Mellitus (%) | 9 (60) |
| High Blood Pressure (%) | 9 (60) |
| Kidney Disease (%) | 4 (26.6) |
| **Hospital Length of Stay** | |
| < 7 days (%) | 8 (53.33) |
| ≥ 7 days (%) | 7 (46.67) |
| **Ventilatory Support** | |
| Invasive (Nose tips, %) | 9 (60) |
| Non-invasive (Intubation, %) | 6 (40) |
| **Hospital Discharge** | |
| Improvement (%) | 9 (60) |
| Fatal cases (death, %) | 6 (40) |


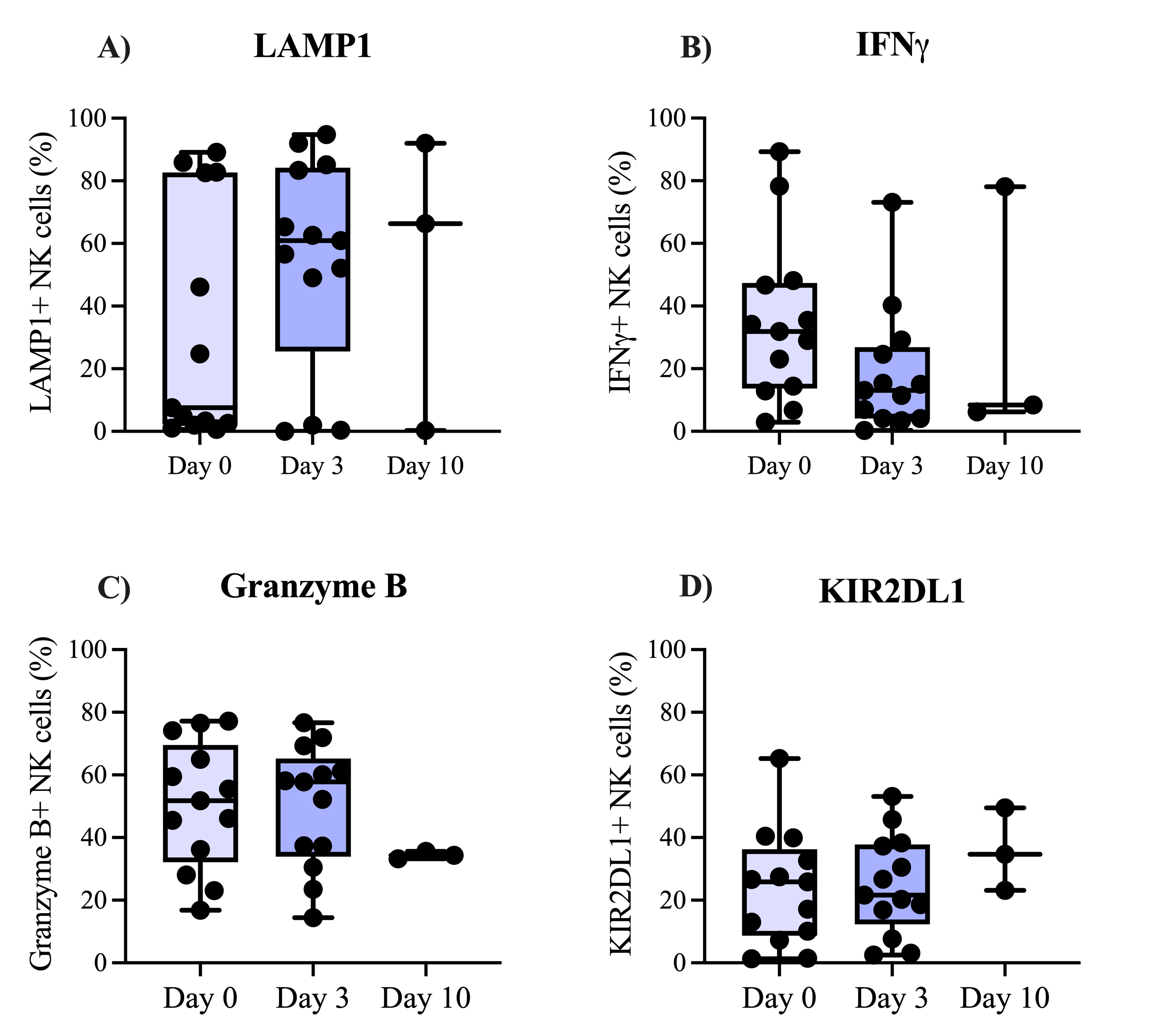


**Figure S3. Dynamics of Activation and Inhibition Markers on NK Cells in Severe COVID-19 Patients During Hospitalization.**  *Expression of LAMP1 (A), IFNγ (B), Granzyme B (C), and KIR2DL1 (D) in NK cell of severe COVID-19 patients in hospital admission, day 3 and 10 of hospital stay. Kruskal-Walli’s test, Dunn`s posthoc. Day 0 (n= 15), day 3 (n= 12) and day 10 (n= 3).*


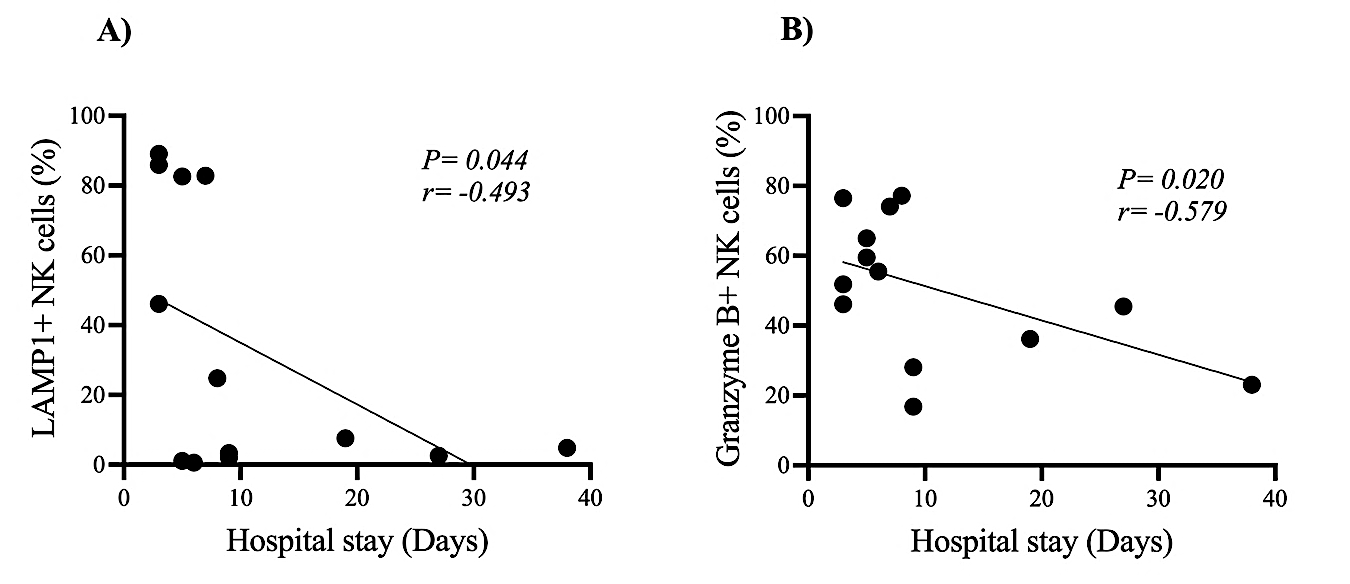


***Figure S4. Correlation Between Activation Markers and Duration of Hospital Stay.*** *Correlation of hospital length of stay (days) with LAMP1 (A) and Granzyme B (B) expression in NK cells from severe COVID-19 patients (n= 13). Spearman’s test, *P< 0.05.*


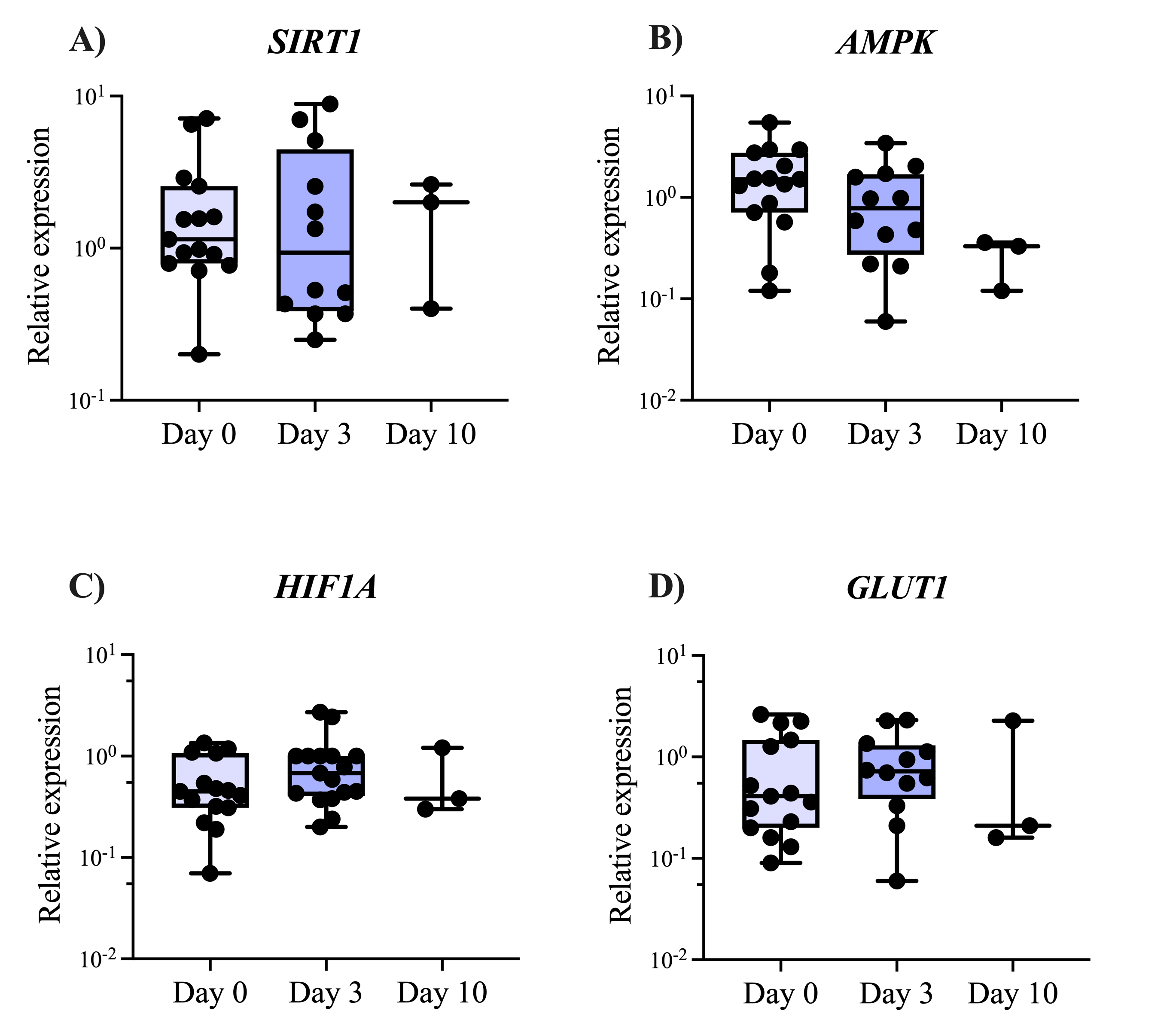


***Figure S5. Expression of metabolic genes in NK cells from severe COVID-19 patients during the hospital stay.*** *Expression of SIRT1 (A), AMPK (B), HIF1A (C), and GLUT1 (D) in NK cell of severe COVID-19 patients in hospital admission, day 3 and 10 of hospital stay. Kruskal-Walli’s test, Dunn`s posthoc. Day 0 (n= 15), day 3 (n= 12) and day 10 (n= 3).*


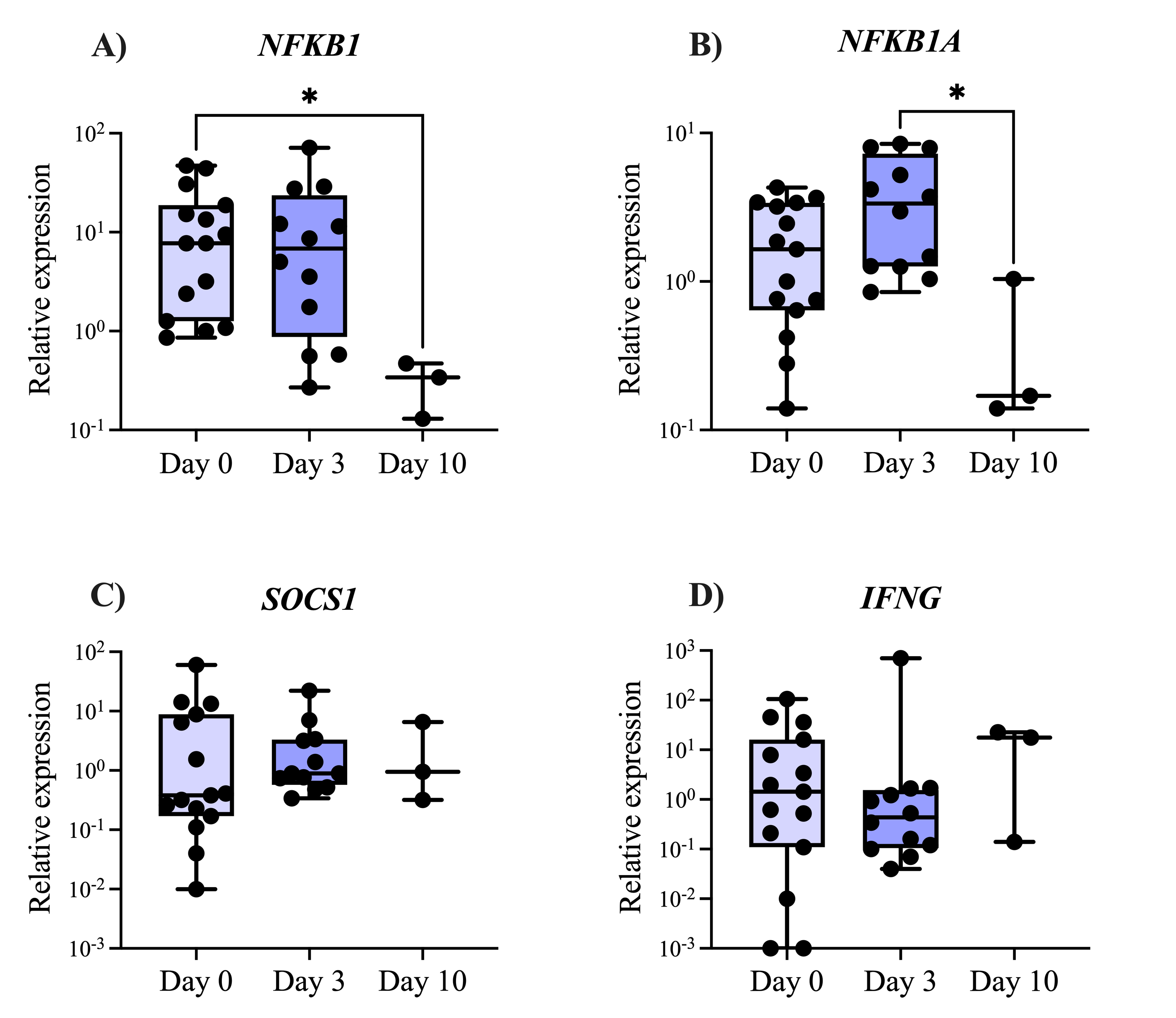


***Figure S6. Expression of inflammatory genes in NK cells from severe COVID-19 patients during the hospital stay.*** *Expression of NFKB1 (A), NFKB1A (B), SOCS1 (C), and IFNG (D) in NK cell of severe COVID-19 patients in hospital admission, day 3 and 10 of hospital stay. Kruskal-Walli’s test, Dunn`s posthoc. Day 0 (n= 15), day 3 (n= 12) and day 10 (n= 3).*

**References**

#

x

| 1. | Thermo Fisher Scientific. Thermo Fisher Scientific. [Online]. [cited 2025 marzo. Available from: <https://www.thermofisher.com/taqman-gene-expression/product/Hs00989291_m1?CID=&ICID=&subtype=>. |
| --- | --- |
| 2. | Thermo Fisher Scientific. Thermo Fisher Scientific. [Online]. [cited 2025 marzo. Available from: <https://www.thermofisher.com/order/genome-database/details/gene-expression/Hs00705164_s1>. |
| 3. | Thermo Fisher Scientific. Thermo Fisher Scientific. [Online]. [cited 2025 marzo. Available from: <https://www.thermofisher.com/order/genome-database/details/gene-expression/Hs00765730_m1>. |
| 4. | Thermo Fisher Scientific. Thermo Fisher Scientific. [Online]. [cited 2025 marzo. Available from: <https://www.thermofisher.com/order/genome-database/details/gene-expression/Hs00355671_g1>. |
| 5. | Ma L, Gao J. Suppression of lncRNA-MALAT1 activity ameliorates femoral head necrosis by modulating mTOR signaling. Arch Med Sci. 2024; 20(2): p. 612-617. |
| 6. | Harmel E, Grenier E, Bendjoudi Ouadda A, El Chebly M, Ziv E, Beaulieu J, et al. AMPK in the Small Intestine in Normal and Pathophysiological Conditions. Endocrinology. 2014; 155(3): p. 873–888. |
| 7. | Xue J, Zhong S, Sun Bm, Sun QF, Hu LY, Pan SJ. Lnc-THOR silencing inhibits human glioma cell survival by activating MAGEA6-AMPK signaling. Cell Death and Disease. 2019; 10(1): p. 1-13. |
| 8. | Aghara H, Chadha P, Mandal P. Mitigative Effect of Graphene Oxide Nanoparticles in Maintaining Gut–Liver Homeostasis against Alcohol Injury. Gastroenterol. Insights. 2024; 15(1): p. 574–587. |
| 9. | Childs-Disney J, Tran T, Vummidi B, Wang ZF, Tellinghuisen T, Disney M. A Massively Parallel Selection of Small Molecule- RNA Motif Binding Partners Informs Design of an Antiviral from Sequence. Chem. 2018; 4(1): p. 2384–2404. |
| 10. | Xiao H, Liu Z. Effects of microRNA‐217 on high glucose‐induced inflammation and apoptosis of human retinal pigment epithelial cells (ARPE‐19) and its underlying mechanism. Molecular Medicine Reports. 2019; 20(1): p. 5125-5133. |
| 11. | Zhang L, Chen J, He Q, Chao Z, Li X, Chen M. MicroRNA‐217 is involved in the progression of atherosclerosis through regulating inflammatory responses by targeting sirtuin 1. Molecular Medicine Reports. 2019; 20: p. 3182-3190. |
| 12. | Deng X, Shang L, Du M, Yuan L, Xiong L, Xie X. Mechanism underlying the significant role of the miR‐4262/SIRT1 axis in children with inflammatory bowel disease. EXPERIMENTAL AND THERAPEUTIC MEDICINE. 2020; 20(1): p. 2227-2235. |
| 13. | Feng C, Jiang H, Yang X, Cong H, Li L, Feng J. GLUT1 Mediates the Metabolic Reprogramming and Inflammation of CCR2+ Monocytes/Macrophages from Patients with DCM. Front. Biosci. 2023; 28(9): p. 223-235. |
| 14. | Liu KX, Chen Q, Chen GP, Huang JC, Huang JF, Re XR, et al. Inhibition of microRNA-218 reduces HIF-1α by targeting on Robo1 in mice aortic endothelial cells under intermittent hypoxia. Oncotarget. 2017; 8(61): p. 104359-104366. |
| 15. | Jhas B, Sriskanthadevan S, Skrtic1 M, Sukhai M, Voisin V, Jitkova Y, et al. Metabolic Adaptation to Chronic Inhibition of Mitochondrial Protein Synthesis in Acute Myeloid Leukemia Cells. Plos One. 2013; 8(3): p. e58367-e8380. |
| 16. | Doganay S, Lee M, Baum A, Peh J, Hwang SY, Yoo JY, et al. Single-cell analysis of early antiviral gene expression reveals a determinant of stochastic IFNB1 expression. Integr. Biol. 2017; 9(11): p. 857-867. |
| 17. | Lin C, Kuzmanovic A, Wang N, Liao L, Ernst S, Penners C, et al. Exceptional Uptake, Limited Protein Expression: Liver Macrophages Lost in Translation of Synthetic mRNA. Adv. Sci. 2025; 12(1): p. 2409729-2409746. |
| 18. | Liu Y, Wang Y, Yang L, Sun F, Wang Y, Zhang GA, et al. The nucleolus functions as the compartment for histone H2B protein degradation. iScience. 2021; 24(1): p. 1-31. |

x
